# Practical courses on advanced methods in macromolecular crystallization: 20 years of history and future perspectives

**DOI:** 10.1107/S1600576724007106

**Published:** 2024-08-30

**Authors:** Petra Havlickova, Jose A. Gavira, Jeroen R. Mesters, Anna Koutska, Barbora Kascakova, Tatyana Prudnikova, Rolf Hilgenfeld, Juan Manuel Garcia-Ruiz, Pavlina Rezacova, Ivana Kuta Smatanova

**Affiliations:** a Department of Chemistry, Faculty of Science University of South Bohemia in Ceske Budejovice Branisovska 1760 37005Ceske Budejovice Czechia; b Laboratorio de Estudios Cristalográficos Instituto Andaluz de Ciencias de la Tierra (CSIC) Avenida de las Palmeras 4 18100Armilla Granada Spain; c Institute of Biochemistry University of Lübeck Ratzeburger Allee 160 23562Lübeck Germany; d Institute of Molecular Medicine University of Lübeck Ratzeburger Allee 160 23562Lübeck Germany; e Donostia International Physics Center Paseo Manuel de Lardizabal 4 20018San Sebastian Spain; f Institute of Organic Chemistry and Biochemistry, AS CR Flemingovo n. 2 166 10Prague 6 Czechia; Wilfrid Laurier University, Waterloo, Ontario, Canada

**Keywords:** crystallogenesis, teaching, hands-on exercises, Czech Republic, FEBS, crystallization

## Abstract

The first Federation of European Biochemical Societies Advanced Course on macromolecular crystallization was launched in the Czech Republic in October 2004. Over the past two decades, the course has developed into a distinguished event, attracting students, early career postdoctoral researchers and lecturers. The course topics include protein purification, characterization and crystallization, covering the latest advances in the field of structural biology. The many hands-on practical exercises enable a close interaction between students and teachers and offer the opportunity for students to crystallize their own proteins. The course has a broad and lasting impact on the scientific community as participants return to their home laboratories and act as nuclei by communicating and implementing their newly acquired knowledge and skills.

## Abstract 2

Since 2004, the University of South Bohemia has been establishing a tradition in protein crystallography through practical crystallization courses organized under the auspices of the Federation of Biochemical Societies.

## 1. Introduction

Macromolecular crystallography is one of the most powerful structural biology techniques. Many fundamental fields of science benefit from this technique, such as chemistry, biology, physics and environmental science. The experimental procedure requires the growth of well diffracting crystals. Knowledge on how to nucleate, grow, analyse, handle and improve crystals is therefore an essential requirement to obtain the necessary X-ray diffraction data that will enable the scientist to determine a three-dimensional arrangement of atoms. Even though basic crystallography principles are theoretically taught at universities, practical training on how to obtain the required crystals is typically missing.

This lack of formal education has already been identified in our community, and the International Union of Crystallography (IUCr) has a permanent Commission on Crystallographic Teaching with its own microsymposia accompanying the regular congress. Still, most of the effort of our community has been focused on the potential of web technologies to better reach students (Kantardjieff, 2010 ▸; Kantardjieff *et al.*, 2010 ▸). Recently, Gražulis *et al.* (2015 ▸) and Dawe *et al.* (2022 ▸) highlighted the role of crystallography as an educational tool taught at different levels. From this series of articles, the need for specialized hands-on workshops or schools to teach crystallization, not only the fundamentals but also the methods and procedures, has been recognized. Under this premise, the Federation of European Biochemical Societies (FEBS) Advanced Crystallization Course was born two decades ago to fill the gap between basic theoretical knowledge and concrete hands-on skills. The FEBS organization provides detailed guidelines for advanced course programmes for course organizers (https://www.febs.org/events/advanced-courses/for-advanced-courses-organizers) with which the course layout must comply. This year, the tenth edition of the FEBS crystallization course took place from the 9th to the 15th of June at the Faculty of Science, University of South Bohemia in Ceske Budejovice, Czech Republic, and included contemporary topics like fragment screening, *in cellulo* protein crystallization and basic cryo-electron microscopy (cryo-EM) principles.

## 2. Historical context

Crystallography is one of the scientific disciplines whose importance has been decisive for the development of chemistry during the last third of the 20th century, due to its contribution to the knowledge of the state of aggregation of matter, in which the components (atoms, ions, molecules) are arranged in an orderly and repetitive manner to form the crystalline state. Over the course of history, many Nobel Prizes (NPs) have been awarded for scientific achievements directly related to, or involving the use of, crystallographic methods and techniques. Starting in 1901 with the NP in Physics awarded to W. C. Röntgen for the discovery of X-rays, to the most recent NP in Physiology or Medicine, 2021, to D. Julius and A. Patapoutian for their discovery of receptors to sense temperature and touch, a total of 33 prizes have been given to crystallography-related investigations and discoveries. Crystallography, when applied to macromolecules, has profoundly influenced biology, biochemistry, physiology and medicine. It is the basis of structural biology and structural genomics, helping us study proteins, nucleic acids, and their complexes such as ribosomes and viruses. Detailed knowledge of the structure of biological macromolecules allows us not only to understand the relationship between structure and function but also to make rational proposals for functional improvement of medication, which is a top priority in today’s bio­medicine.

Unfortunately, there is no widely accessible formal education in this field at either the undergraduate or graduate level. Therefore, the aim of the FEBS Advanced Course on macromolecular crystallization was, from the very beginning, to close this gap of knowledge while exposing the student to focused hands-on practice.

**Figure 1:**
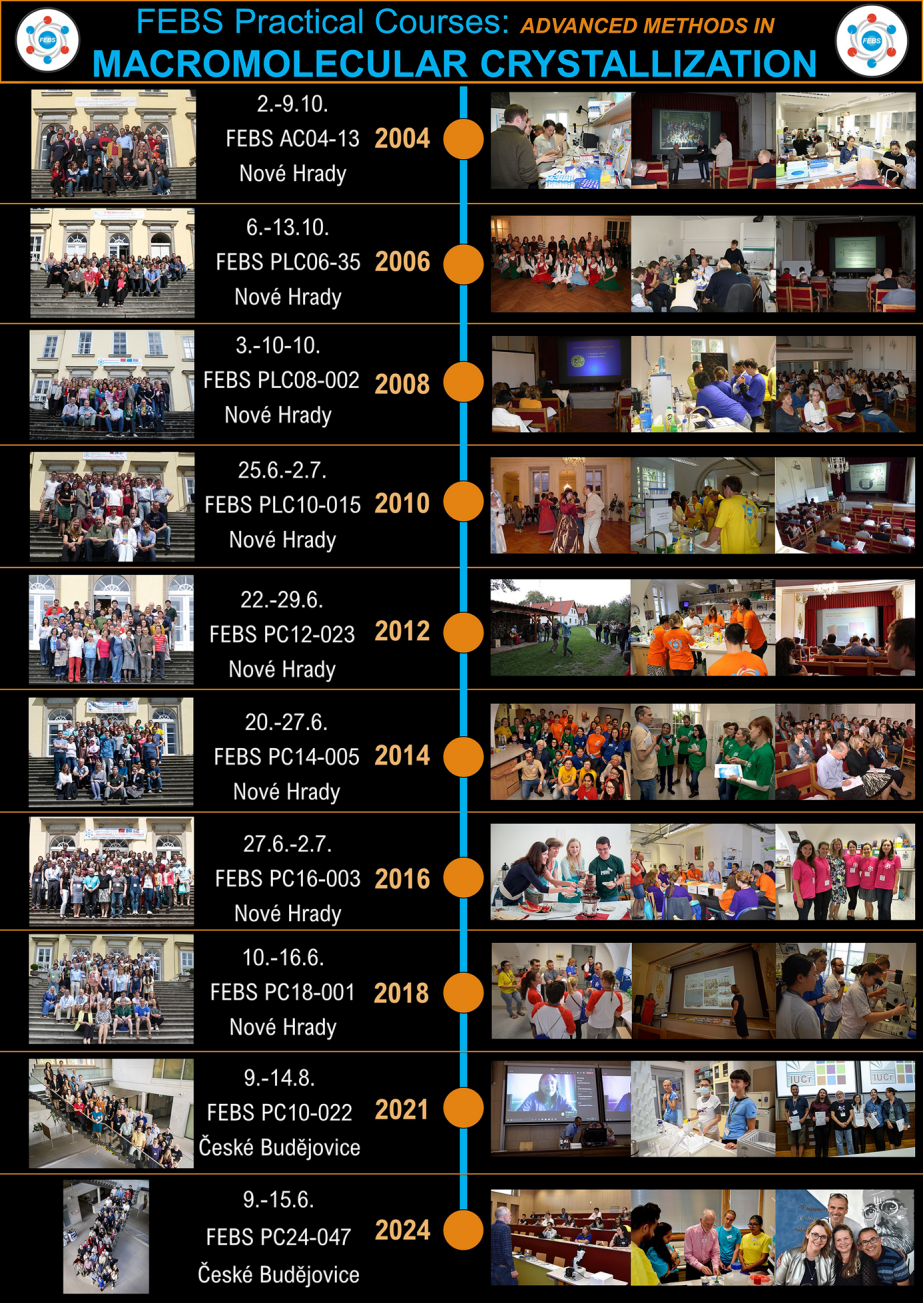
Timeline and snapshots of the FEBS Advanced Course on macromolecular crystallization.

The first concept of this kind of practical crystallization course emerged in the form of the crystallization course (CC) that took place in 2001 and was repeated in 2003 and 2005 in Nové Hrady (Czech Republic) as a locally organized and supported event. In 2004, the course was supported by FEBS and opened to a much broader international community of students and early career researchers. Renamed to ‘Advanced methods in macromolecular crystallization’, it was from thereon organized biennially. Ongoing support from FEBS, along with a strong commitment of the local organizing team and participating international lecturers and tutors, enabled the firm establishment and continuance of the now 20-year-old tradition (Fig. 1 ▸). Despite the difficulties encountered during the COVID pandemic, the FEBS Advanced Crystallization Course took place as one of the rare in-person events in August 2021 (ninth edition), attended by a consistent number of participants. However, the COVID pandemic left its mark and prompted us to postpone the tenth edition by one year.

From 2004 to 2018, the courses were held in the castle of Nové Hrady, located in the south of the Czech Republic (South Bohemian region). This distant and rather remote place allowed all participants to be offered accommodation either on-site or in the vicinity of the castle and thus spend the whole course and free time either informally interacting with each other or working on their scientific project, discussing problems in the laboratory with peers and tutors. Since 2021, the event has been organized at the Faculty of Science of the University of South Bohemia in Ceske Budejovice (Budweis), in the lecture halls and the laboratories of the Structural Chemistry department. The participants are accommodated on the university campus in the student dormitories, close to the venue at the Faculty of Science.

**Figure 2:**
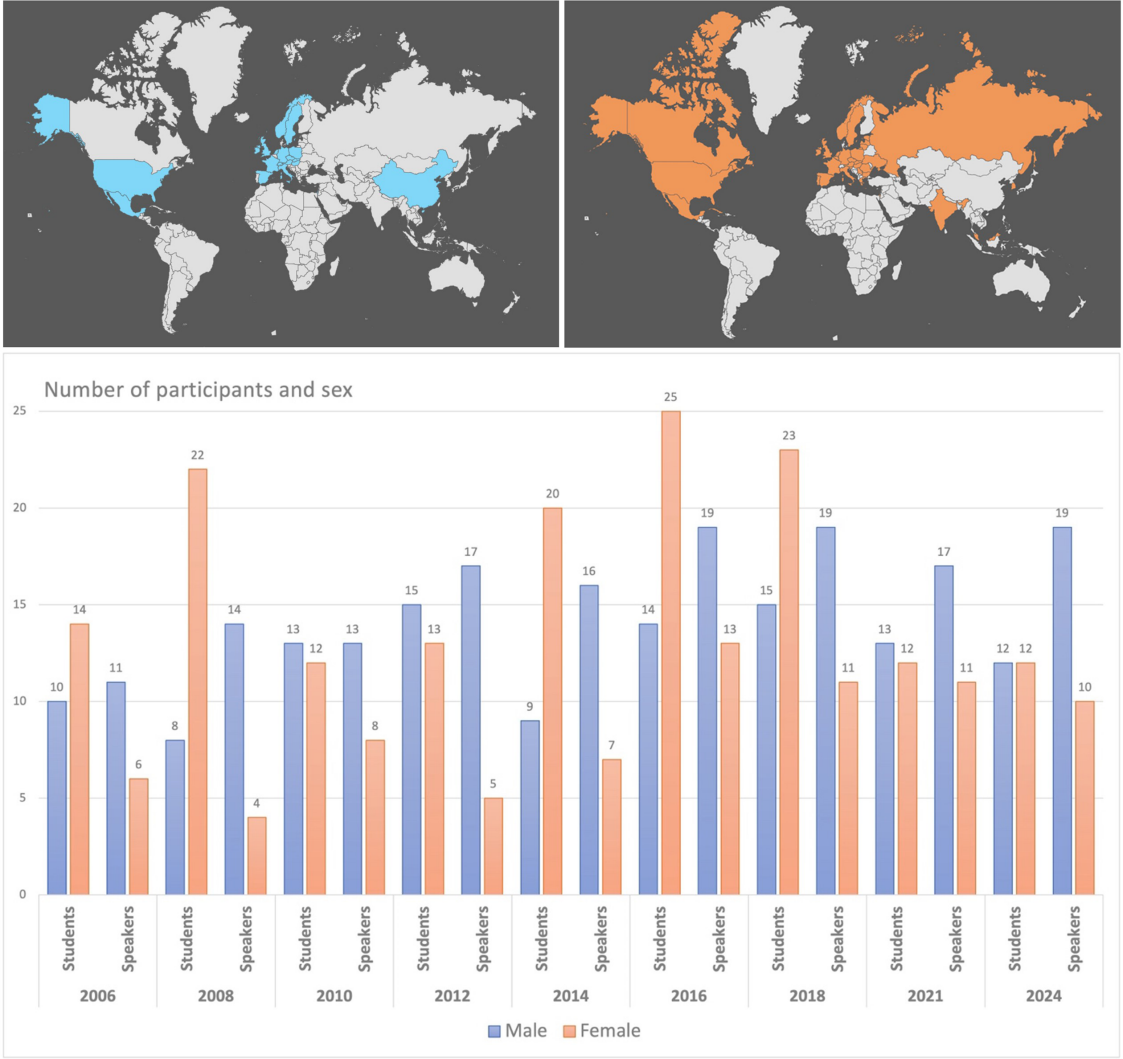
Graphs representing the origin and total number of students and speakers over the past nine editions (2006–2024) of the course. The upper maps show the country of origin of speakers (blue) and students (orange) and the lower graph the total number and sex of students and speakers.

Compared with the very first CC event, the number of applicants almost doubled and increased every year in comparison with the number of available places (Fig. 2 ▸). According to the guidelines for practical FEBS Advanced Courses, the ratio between the number of teachers and students should be close to one or higher, which requires careful selection of the students based on their curriculum vitae, motivation letters and recommendation letters from their supervisors.

## 3. Description of the course

The course not only offers theoretical lectures, covering the fundamental principles of crystallography, sample preparation for crystallization experiments, nucleation and crystal growth, the principles of various crystallization techniques, and experimental strategies for screening and optimizing crystallization conditions, but also incorporates current advancements in structural biology methods, including *in cellulo* crystallization and cryo-EM basics.

**Figure 3:**
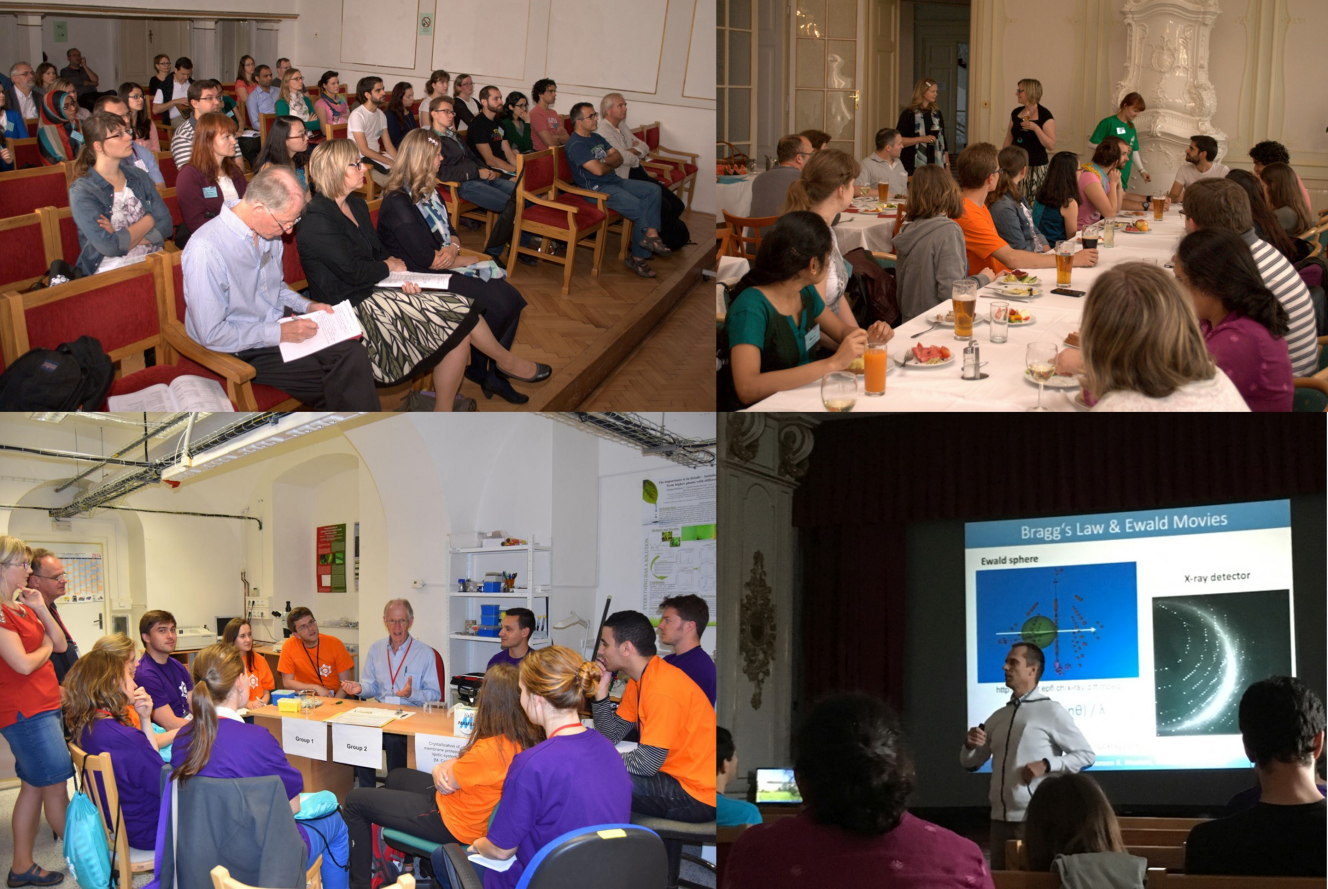
Lectures and practical sessions. From top left to bottom right: participants sitting in the theatre hall during lectures; lunch in the Mirror Hall of the castle; laboratory practical exercise with Martin Caffrey demonstrating the lipid cubic phase; optional evening lecture with Jeroen Mesters explaining Bragg’s law and Ewald’s sphere of reflection.

The FEBS Advanced Crystallization Course is organized as an event lasting six days, starting on Sunday afternoon with some introductory lectures and finishing on Saturday morning, with practical exercises on crystal flash-cooling and data collection. A typical day of the course starts with morning lectures on different crystallization topics given by expert speakers and followed by discussions (Fig. 3 ▸). During the breaks between the lectures or practical exercises, participants can also interact directly with the speakers and discuss their ideas and scientific problems. After lunch, the practical sessions start in the laboratories; the topics usually relate to the morning lectures. Some of the hands-on training sessions are compulsory, some of them are optional, and students can decide according to their preferences and needs. After dinner, the teaching continues with non-compulsory lectures on the theoretical background of crystal structure determination; these optional lectures are not a part of the regular programme of the crystallization course (Fig. 3 ▸).

Two evening sessions are also devoted to the students’ own posters, where students not only present their data but also discuss their difficulties and problems with their peers and the specialists. A scientific committee comprising various specialists also assesses the posters using a standardized scoring sheet, and outstanding ones are awarded poster prizes.

**Figure 4:**
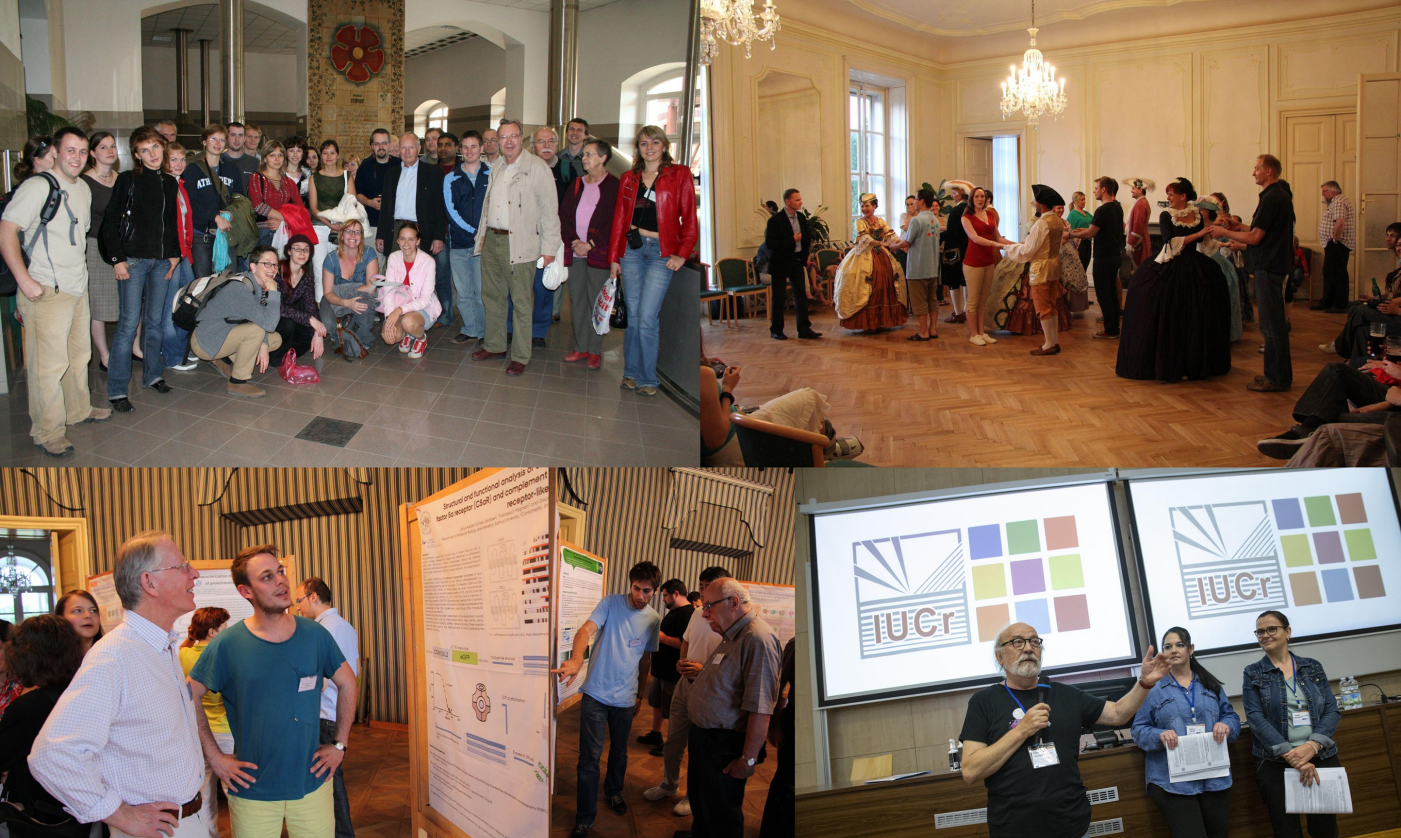
Social activities, poster sessions and poster prizes. From top left to bottom right: visiting the Budweiser Budvar: Czech National Brewery; dancing lesson in the castle; poster session; best poster prizes (awarded in 2021 and 2024 by the IUCr).

Besides the get-together evening sessions, various social events, such as an excursion, historical dancing, concerts or gala dinners, were organized during the courses to let the participants interact in a more informal atmosphere (Fig. 4 ▸).

### 3.1. Topics – theoretical lectures

The FEBS Advanced Crystallization Course was designed to teach the participants a wide range of crystallization and structural biology techniques; the lectures and hands-on practical exercises are given and supervised by experts from all over the world (see Tables S1 and S2 in the supporting information).

Mainly in order to follow the same experimental path required to produce structural information, the first topic is protein purification, including purification of protein complexes and adaptation of the protein sequence, for example, increasing its solubility (Table 1 ▸). How to characterize a protein solution in terms of purity, state of aggregation *etc*. is stressed as a fundamental part of the process prior to crystallization. Before starting with more commonly used crystallization techniques, the concepts of the protein phase diagram, supersaturation, and the thermodynamic principles of crystal nucleation and growth are introduced. The many routes to find initial crystallization conditions are shown and discussed prior to being implemented by the students during the hands-on sessions. From the beginning for each edition, the programme is pre-evaluated and, where needed, adapted following suggestions by tutors and former participants. For example, in the latest edition (2024) we have included a talk on fragment screening. The full list of past and present topics covered during the theoretical lectures is provided in Table 1 ▸ and detailed further in Table S1.

### 3.2. Topics – hands-on practical exercises

**Figure 5:**
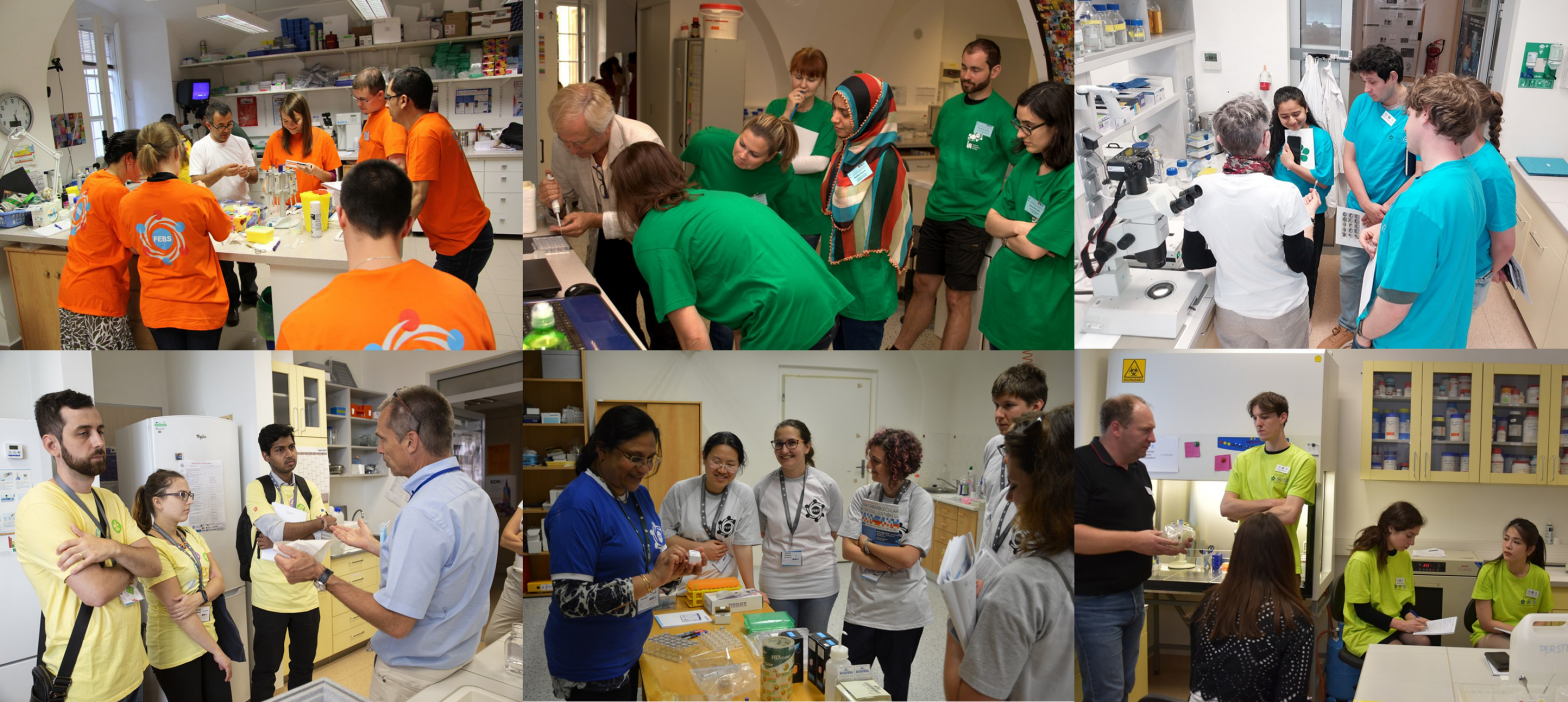
Hands-on training. From top left to bottom right: crystallization in capillaries (Jose A. Gavira); the secret life of crystallization drops (Bernhard Rupp); observation of crystal growth and seeding (Terese Bergfors); crystallizing own proteins (Jeroen R. Mesters); crystallization under oil (Lata Govada); intracellular protein crystallization (Lars Redecke).

The freshly acquired knowledge from the morning lectures is implemented and applied during the four-hour afternoon practical exercises. On average, there were approximately 28 students, who were typically divided into six groups. During the online registration process, students were solicited to bring their own proteins. Students who would like to test their own samples were evenly spread between the different groups to increase their chances to discuss their own problems with the specialist. A T-shirt colour-code was introduced to identify each group composed of approximately four to six students (Fig. 5 ▸). All groups completed all practical exercises according to a prearranged time schedule. In addition, free time slots were available to deepen a particular hands-on skill or to further crystallize students’ own samples as required, choosing one of the techniques offered in the course. A detailed list of past and present topics covered during practical exercises is given in Table S2.

Students bringing their own protein(s) were also required to provide a minimum amount of information such as the final purification column elution profile, Coomassie stained SDS–PAGE gel of the protein and its final concentration, together with a full description of the composition of the solute and storage condition. In addition, model proteins such as lysozyme, thaumatin or xylanase are used to carry out hands-on crystallization experiments.

The students’ proteins were firstly characterized in solution by biophysical methods (*e.g.* dynamic light scattering). The tutors individually discussed the students’ samples and proposed an optimal experimental strategy to obtain crystals. The crystallization trials were observed under a microscope and promising conditions were subsequently optimized using standard and advanced crystallization methods (Fig. 5 ▸). If the desired crystal was obtained, the crystal could be fished and cryocooled in liquid nitro­gen with cryoprotection agents and measured later at the home-source diffractometer or sent to a synchrotron. The direct interaction with the experts, allowing more personalized discussions on specific scientific issues, has resulted in various collaborations and publications: see for example de Wijn *et al.* (2018 ▸, 2019 ▸) and Benedek *et al.* (2019 ▸).

### 3.3. Course organization

**Figure 6:**
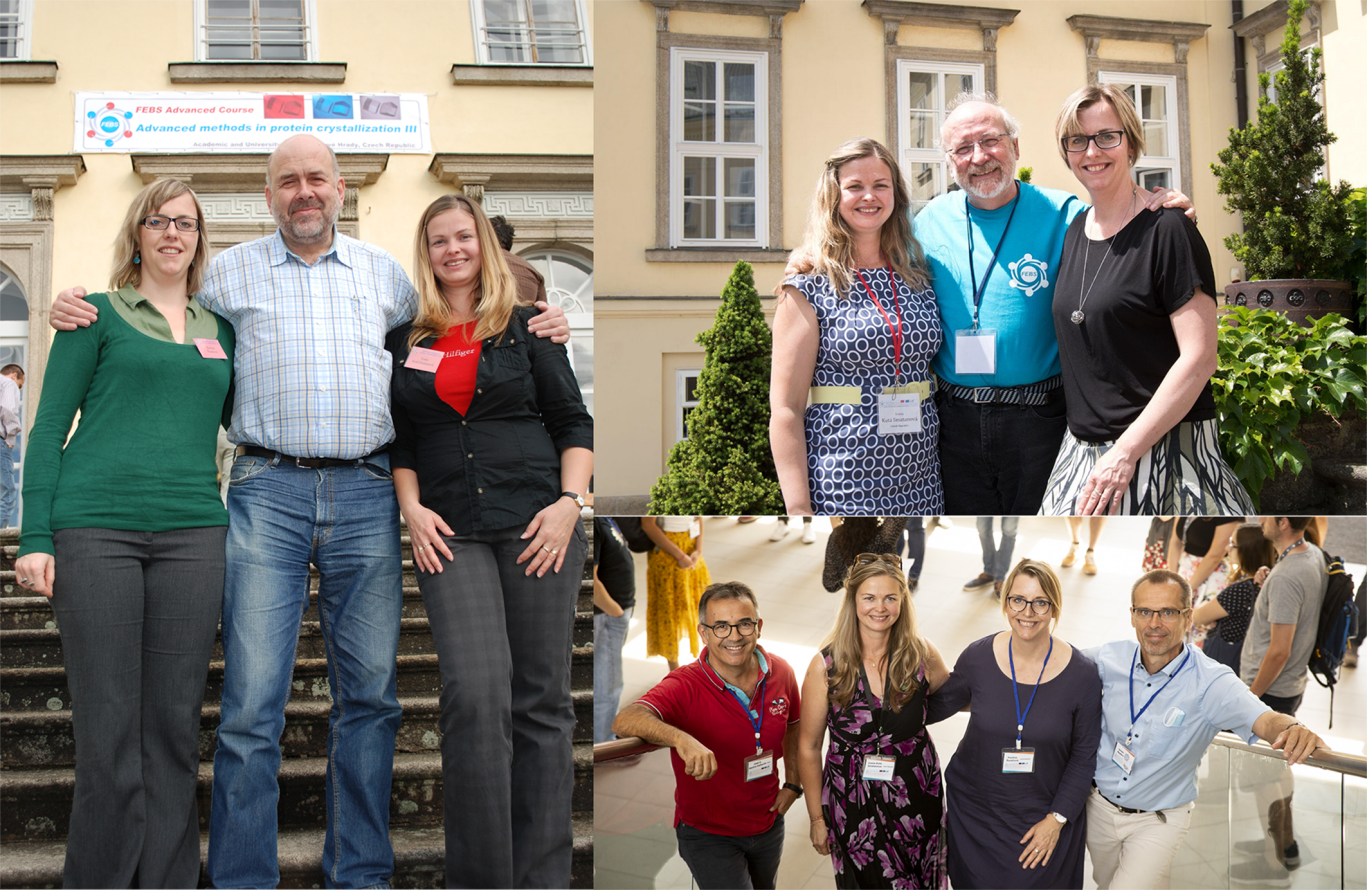
Organizers of the FEBS practical crystallization courses. From left to bottom right: FEBS2004–FEBS2012 (Pavlina Rezacova, Rolf Hilgenfeld and Ivana Kuta Smatanova); FEBS2014–FEBS2018 (Ivana Kuta Smatanova, Juan Manuel Garcia-Ruiz and Pavlina Rezacova); FEBS2021 (Jose A. Gavira, Ivana Kuta Smatanova, Pavlina Rezacova and Jeroen R. Mesters).

Each edition of the course is carefully organized at least one year in advance. The main organizer of the course prepares a proposal for FEBS for the advanced practical course according to the instructions available at https://www.febs.org/events/advanced-courses/for-advanced-courses-organizers. After the selection process, the course organizers may be asked to clarify several points concerning the evaluation of the proposal and filed appendices. Meanwhile, the organizers (Fig. 6 ▸) structure the web pages and course administration, arrange for the communication with all participants, handle the students’ applications and admissions, propose Youth Travel Fund (YTF) grant awardees (see the FEBS web page for further information; https://www.febs.org/events/advanced-courses/for-advanced-courses-organizers), and finalize the programme schedules for lectures and laboratory exercises.

At the earliest opportunity, potential sponsors are contacted to seek their support for the course. Sponsors have the option to select the way in which they would like to contribute. Typically, companies provide consumables for hands-on practical exercises, support coffee breaks or evening activities (refreshments during evening lectures), or cover registration fees for individual students. All forms of sponsorship are highly appreciated. The sponsor’s logos and advertisements are prominently displayed on all course materials, on the web page, and in the lecture hall and poster display area.

With the support of FEBS and the University of South Bohemia, along with the strong commitment of the local organizing team and the participating international lecturers and tutors, we have been able to keep the registration fee low for an extended period, especially in comparison with other similar courses worldwide.

The collected abstracts of all lectures, laboratory exercises and poster presentations are published in a special issue of the Bulletin of Czech and Slovak Crystallographic Association, *Materials Structure in Chemistry, Biology, Physics and Technology* [ISSN 1211–5894 (print), ISSN 1805–4382 (online)].

### 3.4. Participants

**Figure 7:**
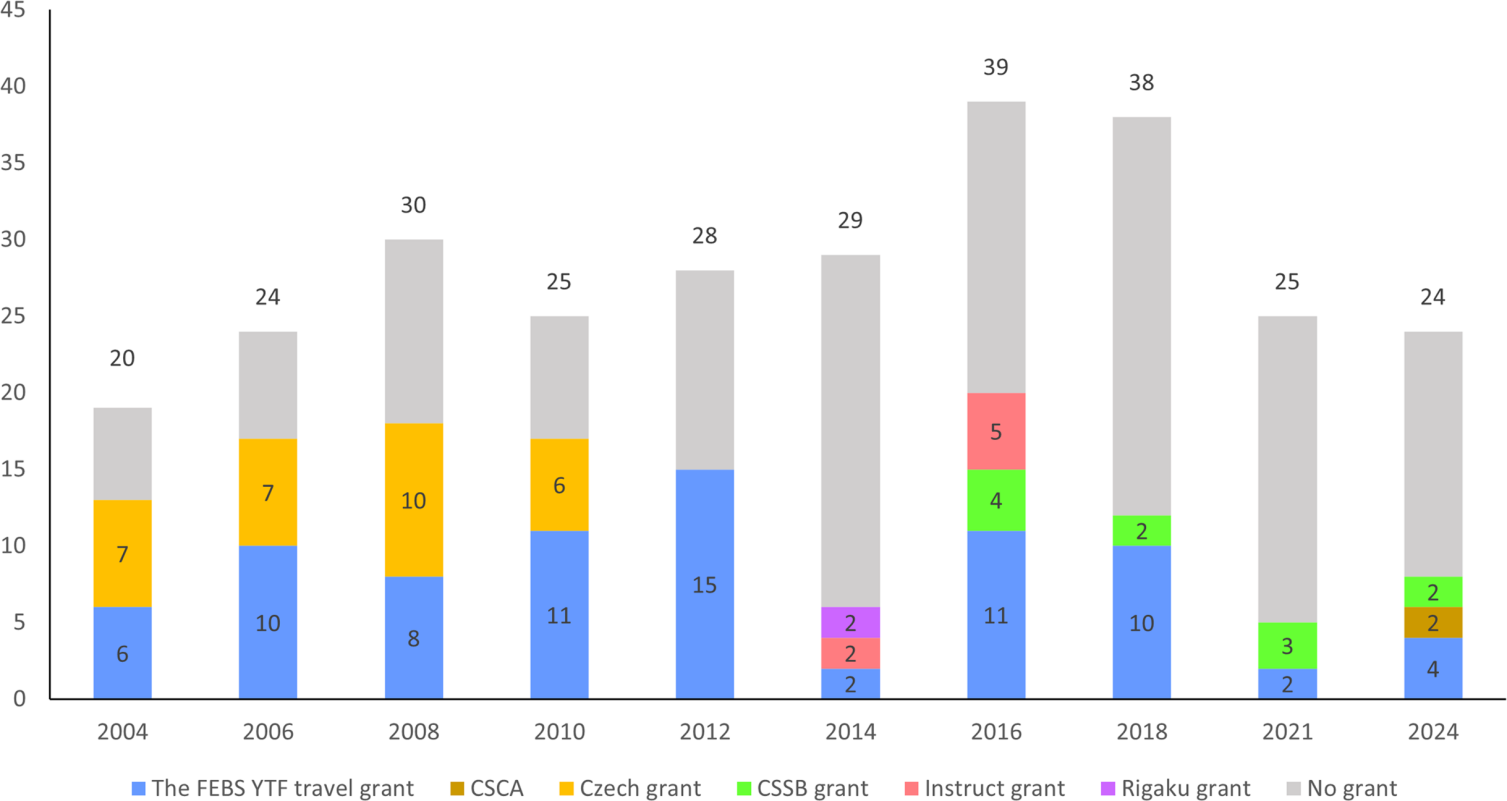
Number of grants provided to students by institutions, associations and companies.

The number of students participating in FEBS Advanced Courses is generally limited to 24, with a ratio of tutors/teachers to students of 1:2, in agreement with the FEBS criteria. The maximum capacity of the Laboratory of Structural Chemistry is 30 students, and with prior consent of the FEBS Advanced Courses (AC) committee the number of students admitted to the CCs was frequently increased to on average 28 students (Fig. 7 ▸). The steady interest of students for this course over the past 20 years has stimulated and convinced us as organizers, tutors and lecturers to continue to offer this course.

Applicants such as PhD students and junior postdoctoral scientists fulfilling the required criteria have the possibility to apply for a FEBS YTF grant supporting their participation at the FEBS course. In addition, a transcontinental YTF award can be offered for the participation of young scientists from outside the FEBS area. Several societies and associations have also supported students at the courses, such as the Czech and Slovak Crystallographic Association (https://xray.cz) and the Czech Society for Structural Biology (https://cssb.structbio.org/) (Fig. 7 ▸).

### 3.5. Course evaluation

During the closing ceremony, participants receive their certificate of participation in exchange for the filled FEBS evaluation questionnaire. In this questionnaire, participants rate (either excellent, good, adequate, poor or inadequate) the programme organization, programme quality, scientific training by the speakers and tutors, subject state of the art, time for discussions, balance between hands-on training and lectures, duration of the course, location and accommodation, and quality of facilities (lecture hall and laboratories) including technical equipment. In addition, space is provided for suggestions for improvements of future courses and for additional comments.

**Figure 8:**
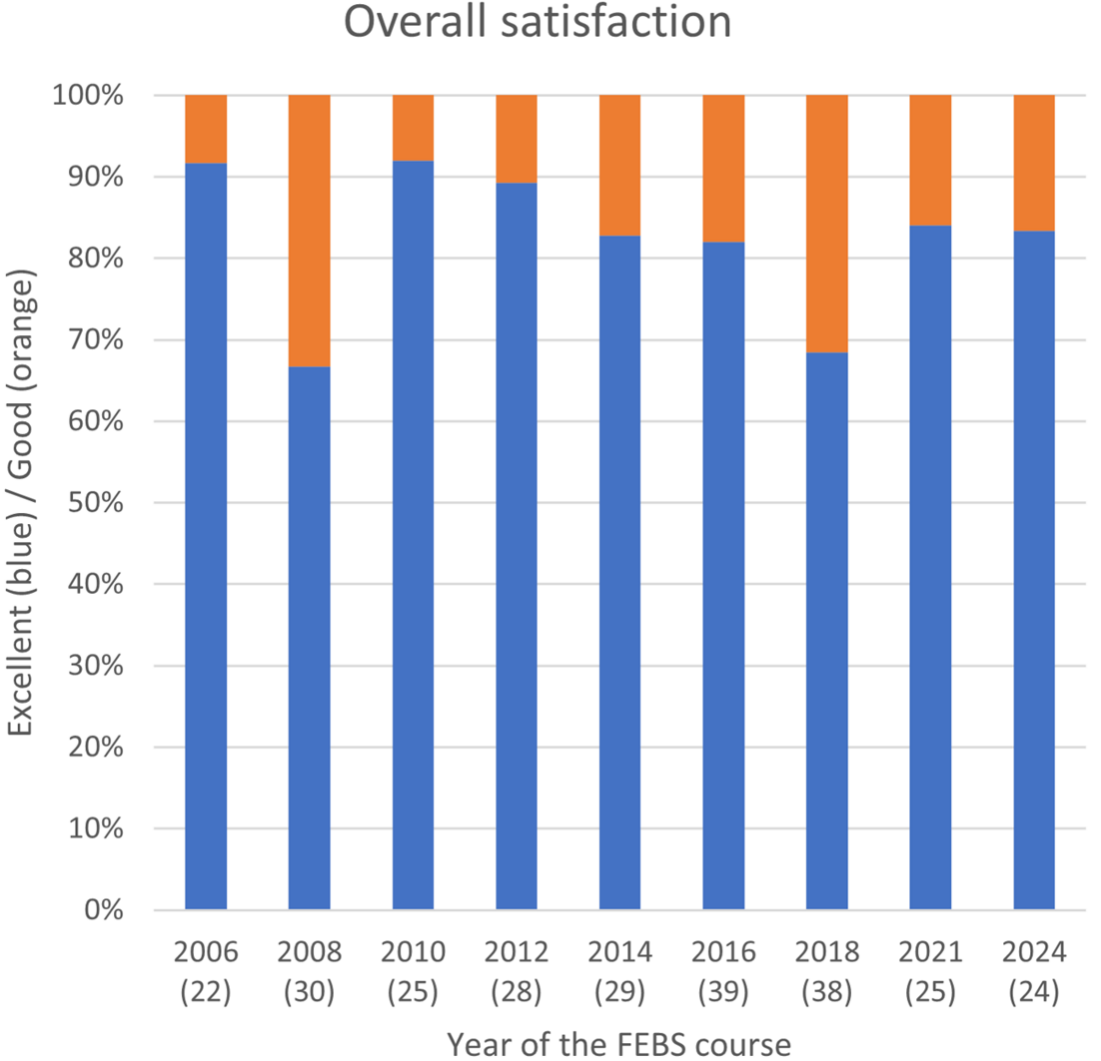
Summary of the averaged scores by year of the FEBS evaluation questionnaire. The number of students that evaluated the courses is given in brackets.

Overall, the participants rated most issues surveyed as excellent or good, with only some solitary/personal adequate to inadequate ratings on one or another single item. The overall averaged rating over all participants is shown in Fig. 8 ▸.

## 4. Future perspectives

Conventional X-ray diffraction techniques, along with new technologies in X-ray free-electron lasers, neutron diffraction, serial crystallography *etc*., have diversified the number of strategies for determining atomic models of macromolecules and macromolecular assemblies while expanding the requirements for crystalline samples. Nowadays, samples are required to be either a single crystal or a slurry of thousands of nanocrystals of homogeneous size, in the size range of nanometres to millimetres. Samples are characterized either at room temperature or under cryogenic conditions. In any case, the need for a crystal sample still requires the right crystallization conditions, which even today are mainly found by a trial-and-error approach. Although artificial intelligence (AI) may soon contribute to this process, we still require more fundamental knowledge on the mechanisms involved in protein crystal growth, as today’s overall knowledge is still insufficient to predict the behaviour of a particular protein in solution. Therefore, spreading basic knowledge on protein crystallization at dedicated practical courses for young scientists and showing how both traditional and advanced methods can be used to screen the phase diagram have not lost their importance, and will remain an important task in the near future.

Due to the course’s ongoing popularity and financial support from the FEBS AC committee, this year the tenth-anniversary edition has been held. This CC is unique in the field of macromolecular crystallization thanks to its practical focus and substantial discussions on developments and methodologies, its coverage of a wide range of structural methods, and, most importantly, its continuing acceptance by the students. Building on this success, we plan to continue to offer the course, maintaining the established organizational structure while incorporating relevant topics beyond the recently assimilated *in cellulo* crystallization, fragment screening, cryo-EM basics, or the role of *AlphaFold* and AI in future development. The aim of the course has always been to educate and motivate young scientists to undertake approaches that are more rational to the crystallization of macromolecules and to combine numerous techniques to tackle their structural problems.

## 5. Conclusions

The legacy of these CCs, which we call advanced because they included unusual techniques and methods, lies not only in their temporal persistence but also in the lasting impact they have had in shaping the research trajectories of a large group of students, industrial applications and academic activities in the wide-ranging field of crystallization. The continuation of these courses serves as a beacon, guiding and inspiring the next generation of scientists and engineers towards new frontiers of discovery and innovation.

Despite the divergent levels of impact on the students’ academic development, student participation in these events has formed a cohesive community that transcends individual courses and extends its influence on other educational programmes, including the International School of Biological Crystallization (Granada) and the FEBS practical and lecture course named ‘Biomolecules in action’ (Budweis and Ham­burg). Additionally, the community involves its teachers and tutors in global conferences such as the International Conference on the Crystallization of Biological Macromolecules and collaborates with entities such as the IOBCr (International Organization for Biological Crystallization) (https://en.wikipedia.org/wiki/International_Organization_for_Biological_Crystallization). This strengthens networking and knowledge and experience transfer within a wider scientific community.

## Supplementary Material

Supporting information. DOI: 10.1107/S1600576724007106/dv5018sup1.pdf

## Figures and Tables

**Table 1:** Topics offered at the FEBS practical crystallization courses

Fundamentals	Nucleation; crystal growth mechanism; phase diagrams
Sample preparation	Construct design; sequence adaptation; entropy reduction; expression; purification
Sample characterization	Dynamic light scattering/static light scattering/differential scanning fluorimetry; microscale thermophoresis; UVEX crystal imaging systems; ultracentrifugation
Crystallization methods	Vapour diffusion, hanging and sitting drop; crystallization under oil; high-throughput methods; lipidic cubic phase and micelles; dialysis and temperature control methods; complexes, protein–protein/protein–nucleic acid
Advanced methods	Liquid diffusion methods: capillaries/microfluidics; fluorescent trace labelling; seeding/microseed matrix screening; ionic liquids, nano/microcrystals for X-ray free-electron lasers; *in cellulo* crystallization; fragment screening
Complementary methods	Small-angle X-ray scattering/small-angle neutron scattering; NMR; cryo-EM
Other methods	Crystal handling; diffraction measurement; publication of scientific results
