# Supplementary material for: Practical courses on advanced methods in macromolecular crystallization: 20 years of history and future perspectives
Source: J Appl Crystallogr. 2024 Aug 30;57(Pt 5):1609–17. doi: 10.1107/S1600576724007106 (PMC11460401; doi:10.1107/S1600576724007106)
Supplement: Supplementary file 1 [file j-57-01609-sup1.pdf]

JOURNAL OF APPLIED CRYSTALLOGRAPHY

Volume 57 (2024)

Supporting information for article:

Practical courses on advanced methods in macromolecular crystallization: 20 years of history and the future perspective

Petra Havlickova, Jose A. Gavira, Jeroen R. Mesters, Anna Koutska, Barbora Kascakova, Tatyana Prudnikova, Rolf Hilgenfeld, Juan Manuel Garcia-Ruiz, Pavlina Rezacova and Ivana Kuta Smatanova

Table S1 List of topics and their instructor(s) for each FEBS Advanced Course on macromolecular crystallization.

Topic/Year of FEBS course	2004	2006	2008	2010	2012	2014	2016	2018	2021	2024	Names of speakers
**Protein samples for crystallization**											**Rezacova, Pavlina** (Prague, C. Republic)
**Alternative crystallization techniques**											**Kuta Smatanova, Ivana** (Ceske Budejovice)
**Conventional methods**											**Mesters, Jeroen R.** (Lübeck, Germany)
**X-ray diffraction Theory** (evening lectures)											**Mesters, Jeroen R.** (Lübeck, Germany)
**Crystallization drops evaluation**											**Bergfors, Terese** (Uppsala, Sweden); **Betzel, Christian** (Hamburg, Germany)
**Seeding strategies**											**Bergfors, Terese** (Uppsala, Sweden); **Shaw-Stewart, Patrick** (Berkshire, UK); **Kolek, Stefan** (Berkshire, UK)
**Nucleation and crystal growth**											**García-Ruiz, JuanMa** (Granada, Spain); **Velikov, Peter G.** (Houston, TX, US); **Gavira, José A.** (Granada, Spain);
**Capillary Counter diffusion**											**Gavira, José A.** (Granada, Spain); **García-Ruiz, JuanMa** (Granada, Spain)
**DLS**											**Betzel, Christian** (Hamburg, Germany); **Dierks, Karsten** (Hamburg, Germany); **Klupsch, Thomas** (Jena, Germany)
**Protein modification for crystallization**											**Urbanikova, Lubica** (Bratislava, Slovakia)
**Protein expression and purification**											**Grandori, Rita** (Milano, Italy); **Janda, Lubos** (Brno, Czech Republic); **Pineda Molina, Estela** (Granada, Spain); **Chaloupkova, Radka** (Brno, Cz. Republic); **Martinez-Rodriguez, Sergio** (Granada, Spain)

**Crystal manipulation and handling**											**Gavira, José A.** (Granada, Spain)
**DNA and RNA crystallization**											**Giege, Richard** (Strasbourg, France)
**Additives in crystal nucleation and growth**											**Hilgenfeld, Rolf** (Lübeck, Germany); **Dohnalek, Jan** (Prague, Czech Republic)
**Solution properties, phase diagram**											**Drenth, Jan** (Groningen, The Netherlands)
**Protein refolding**											**Lange, Christian** (Halle, Germany)
**Membrane protein crystallization, lipid cubic phase**											**Caffrey, Martin** (Dublin, Ireland); **Luecke, Hartmut** (Lisbon, Portugal); **Saenger, Wolfram** (Berlin, Germany); **Zajonc, Dirk M.** (La Jolla, CA, US)
**Limited proteolysis**											**Carey, Janet** (Princeton, NJ, US); **Basquin, Jerome** (Munich, Germany)
**Diffraction quality of crystals**											**Smith, Vernon** (Karlsruhe, Germany)
**In-vitro folding**											**Rudolph, Rainer** (Halle, Germany)
**Microfluidic chips**											**Sauter, Claude** (Strasbourg, France)
**Microseeding, random microseeding, crystal optimization**											**Shaw-Stewart, Patrick** (Berkshire, UK); **Kolek, Stefan** (Berkshire, UK); **D´Arcy, Alan†** (Allschwil, Switzerland); **Marsh, May** (Villigen, Switzerland)
**Unconventional crystallization techniques**											**Chayen, Naomi E.** (London, UK); **Govada, Lata** (London, UK)
**PDB database**											**Schneider, Bohdan** (Prague, Cz. Republic)
**Publication of scientific results**											**Einspahr, Howard** (Lawrenceville, US)
**Neutron crystallography**											**Budayova-Spano, Monika** (Grenoble, France); **Ng, Joseph D.** (Huntsville, AL, US)
**Fluorescent labeling, Ionic liquid**											**Pusey, Marc, L.** (Huntsville, AL, US); **Tarver, Crissy L.** (Stanford, CA, US)
**Crystallization of protein complexes, ligand interactions**											**Nemcovicova, Ivana** (Bratislava, Slovakia)

**Principles of protein crystallization**											**Rupp, Bernhard** (San Diego, CA, US; Innsbruck, Austria)
**Evaluation of crystallization trials using UV**											**Gordon, James** (Suffolk, UK); **Bruystens, Jessica** (Suffolk, UK)
**Crystallization of protein – nucleic acid complexes**											**Biertumpfel,Christian** (Martinsried, Germany)
**Statistical Crystallization**											**Da-Chuan, Yin** (Xi´an, China)
**SAXS**											**Roessle, Manfred** (Lübeck, Germany)
**Complex view into structure**											**Ventachalam, K. V.** (Ft. Lauderdale, US)
**Controlling size and shape of protein crystals**											**Moreno, Abel** (Mexico City, Mexico)
**XFEL**											**Roessle, Manfred** (Lübeck, Germany); **Fromme, Petra** (Tempe, AZ, US); **Cohen, Aina E.** (Stanford, CA, US); **Pachl, Petr** (Prague, Czech Republic)
**Cryo-EM**											**Cunha, Eva** (Lisbon, Portugal); **Degtjarik, Oksana** (Leeds, UK); **Iermak, Iuliia** (Munich, Germany)
**AFM**											**Novotny, Dusan** (Brno, Czech Republic)
**Intracellular crystallization**											**Redecke, Lars** (Lübeck, Germany)
**AlphaFold**											**Ng, Joseph D.** (Huntsville, AL, US)
**Synthetic macrocycles**											**Crowley, Peter** (Galway, Ireland)
**From target structures to drugs**											**Brancale, Andrea** (C. Republic, Prague)
**Crystallographic fragment-screening**											**Weiss, Manfred** (Berlin, Germany)
**Advancements in imaging technologies**											**Kobidova, Barbora** (Prague, Cz. Republic)

Table S2 Practical exercises, ordered by topic, and with the associated tutor(s).

**Topic/Year of FEBS course**	**2004**	**2006**	**2008**	**2010**	**2012**	**2014**	**2016**	**2018**	**2021**	**2024**	**Names of tutors**
**Evaluation of crystallization drops**	■	■	■	■	■	■	■	■	■	■	**Bergfors, Terese (Uppsala, Sweden); Betzel, Christian (Hamburg, Germany)**
**Seeding**	■	■	■	■	■	■	■	■	■	■	**Bergfors, Terese (Uppsala, Sweden)**
**Crystal handling, testing, cryocooling**	■	■	■	■	■	■	■	■	■	■	**Rezacova, Pavlina (Prague, Czech Republic); Brynda, Jiri (Prague, Czech Republic); Pachl, Petr (Prague, Czech Republic); Gavira, José A. (Granada, Spain)**
**DLS**	■	■	■	■	■	■	■	■	■	■	**Betzel, Christian (Hamburg, Germany); Dierks, Karsten (Hamburg, Germany)**
**Capillary protein crystallization**	■	■	■	■	■	■	■	■	■	■	**Gavira, José A. (Granada, Spain)**
**Conventional crystallization techniques**	■	■	■	■	■	■	■	■	■	■	**Mesters, Jeroen R. (Lübeck, Germany)**
**Crystallization of own proteins**	■	■	■	■	■	■	■	■	■	■	**Mesters, Jeroen R. (Lübeck, Germany); Urbanikova, Lubica (Bratislava, Slovakia)**
**Membrane protein crystallization**		■	■	■	■	■	■	■	■	■	**Caffrey, Martin (Dublin, Ireland)**
**Limited proteolysis**		■		■							**Carey, Janet (Princeton, NJ, US)**

**Diffraction experiments**											**Smith, Vernon (Karlsruhe, Germany)**
**Crystallization under oil**											**Chayen, Naomi E. (London, UK);**<br>**Govada, Lata (London, UK)**
**Publication of scientific results**											**Einspahr, Howard (Lawrenceville, NJ, US)**
**Microseeding**											**Shaw-Stewart, Patrick (Berkshire, UK);**<br>**Kolek, Stefan (Berkshire, UK);**<br>**Pachl, Petr (Prague, Czech Republic)**
**Crystallization in microfluidic chips**											**Sauter, Claude (Strasbourg, France)**
**Crystals for neutron diffraction**											**Ng, Joseph D. (Huntsville, AL, US)**
**Trace fluorescent labelling**											**Pusey, Marc, L. (Huntsville, AL, US);**<br>**Tarver, Crissy L. (Stanford, CA, US)**
**The secret life of your crystallization drop**											**Rupp, Bernhard (San Diego, CA, US; Innsbruck, Austria)**
**Crystallization of protein-nucleic acid complexes**											**Biertumpfel, Christian (Martinsried, Germany)**
**Evaluation of crystallization trials with the UVEX microscope**											**Gordon, James (Suffolk, UK);**<br>**Bruystens, Jessica (Suffolk, UK)**
**Single particle cryo-EM**											**Cunha, Eva (Lisbon, Portugal);**

											**Gardian, Zdenko (Ceske Budejovice, Czech Republic); Iermak, Iuliia (Munich, Germany)**
**Controlling the size and the shape of protein crystals**											**Moreno, Abel (Mexico City, Mexico)**
**Tools for remote access crystallography**											**Cohen, Aina E. (Stanford, CA, US)**
**MST, nanoDSF**											**Kania, Pavel (Krakow, Poland);**<br>**Nowak, Jakub (Krakow, Poland)**
**Experimental phasing**											**Basquin, Jerome (Munich, Germany)**
**Intracellular protein crystallization**											**Redecke, Lars (Lübeck, Germany)**
**AlphaFold**											**Ng, Joseph D. (Huntsville, AL, US)**
**Soaking and co-crystallization**											**Kascakova, Barbora (Ceske Budejovice, Czech Republic)**
